# Simple Procedure to Compute the Inductance of a Toroidal Ferrite Core from the Linear to the Saturation Regions

**DOI:** 10.3390/ma6062452

**Published:** 2013-06-17

**Authors:** Rosa Ana Salas, Jorge Pleite

**Affiliations:** Electronic Technology Department, Higher Polytechnic School, Carlos III University of Madrid, Avda. de la Universidad, 30, 28911, Leganés, Madrid, Spain; E-Mail: pleite@ing.uc3m.es

**Keywords:** 2D Finite Element Analysis, soft ferrite cores, nonlinear inductors

## Abstract

We propose a specific procedure to compute the inductance of a toroidal ferrite core as a function of the excitation current. The study includes the linear, intermediate and saturation regions. The procedure combines the use of Finite Element Analysis in 2D and experimental measurements. Through the two dimensional (2D) procedure we are able to achieve convergence, a reduction of computational cost and equivalent results to those computed by three dimensional (3D) simulations. The validation is carried out by comparing 2D, 3D and experimental results.

## 1. Introduction

**Figure 1:**
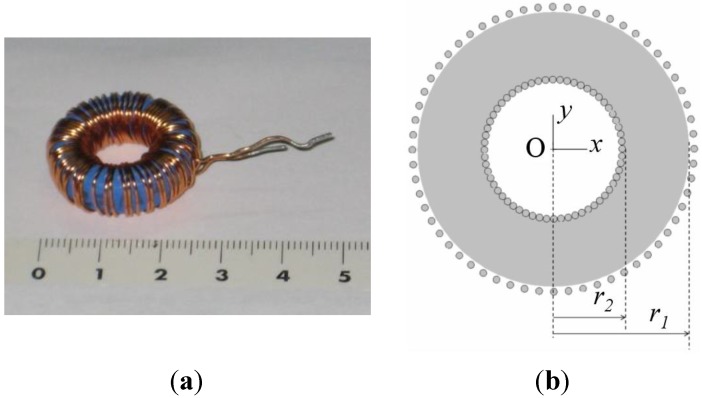
(**a**) Real inductor; (**b**) 2D model.

Ferrites are of great interest for power electronics due to their low power losses [1,2] and they form an essential part of inductors and transformers used in their main applications areas [3,4,5]. Therefore, it is necessary to investigate and model the magnetic properties and the nonlinear behavior of ferrites, which exhibit saturation, hysteresis and power losses. These effects and the great variety of core geometries (e.g., E, RM, POT, toroidal) and other parameters, such as the number of turns, make it difficult to obtain a single model that is both simple and precise. Of all the geometries, the toroidal core (Figure 1a) is the most studied in current literature [6,7,8,9,10,11,12]. Nevertheless, despite these publications there are not enough studies that calculate parameters to be used in circuit simulators, such as inductances in all working regions of the ferrite (linear, intermediate and saturation), geometries and number of turns. In this context, our previous publications have focused on 2D Finite Element Analysis procedures for RM and POT ferrite cores [13,14]. In addition, in the case of the RM core, we have shown an application of our procedures in a commercial circuit simulator [15].

In this paper we focus on the modeling of ferrite inductors with toroidal cores and their nonlinear behavior. We present a specific procedure to compute the inductance of an inductor with a toroidal ferrite core.

We present the 2D model (cross-section of the real inductor) and then we study, by numerical simulations and experimental measurements, if this model is suitable for the simulation of ferrite cores in the linear, intermediate and saturation regions. To do so, we compare 3D and 2D results with experimental measurements. The validation is carried out based on convergence and computational cost, spatial distribution of the magnetic fields and flux and inductance curves. At the same time we present preliminary studies of convergence and computational cost in 2D and 3D, showing the reduction of the computational cost and the similarity of the results.

The outline of the paper is as follows. In Section 2, we present and describe the specific Finite Element procedure to calculate the inductance. We also describe in detail the measuring procedure we use to validate the procedure and to calculate the input parameters. In Section 3 we provide results obtained in the preliminary study of the convergence and computational cost, and we show the results of the magnetic flux, inductance and magnetic fields. Finally, Section 4 briefly summarizes our main conclusions.

## 2. Finite Element Procedure to Compute the Nonlinear Inductance

We begin by specifying the notation that will be used in the rest of this paper. We use **B** and **H** (in bold) to denote the magnetic field vectors and |**B**| and |**H**| to represent the moduli of the magnetic field vectors. We also use *Φ* to indicate the magnetic flux, *L* the inductance of the inductor and *I* the DC excitation current value that flows through the inductor. The aim of the procedure is to compute the *L-I* curve. To do this, we propose a procedure that combines experimental measurements with the use of Finite Element Analysis in 2D. We also carry out 3D simulations in order to compare the results. The procedure involves three steps: premodeling, simulation and postmodeling, where we use the new domain designed for the 2D simulations.

In the *premodeling* step, we measure the magnetic characteristics of the ferrite (the *B-H* curve measured with a two-winding toroidal core with the same core material as the inductor to be studied). This curve is one of the input parameters for the 2D and 3D simulations. In this same step we also obtain the *L-I* curve experimentally in order to validate the results of the program by comparing these measurements. To do so, we take experimental measurements with a DC power supply and an electronic fluxmeter.

In order to obtain the *B-H* curve, we build a two-winding toroidal transformer with the same material as the studied ferrite and measure the *Φ-I* curve for current values from 0 to core saturation. We measure the flux in the secondary winding with the electronic fluxmeter and provide the current with the DC power supply. *B* and *H* values were computed as:

(1) $$B\approx \frac{1}{N_{s}A_{e}}\int v\;dt=\frac{\Phi }{N_{s}A_{e}}$$

(2) $$H\approx \frac{N_{p}I}{\ell_{e}}$$

where *N_p_* and *N_s_* are the number of turns of the primary and secondary winding respectively; *A_e_* is the effective cross-sectional area of the ferrite core; and *ℓ_e_* is the effective magnetic path length. These parameters are detailed by the manufacturer in the catalog.

In order to obtain the *L-I* curve, we build a two-winding transformer with the same core as the studied one and measure the *Φ-I* curve for current values from 0 up to core saturation. From this, we derive the *L-I* curve as:

(3) $$L\left(I\right)=\frac{d\Phi }{dI}$$

**Figure 2:**
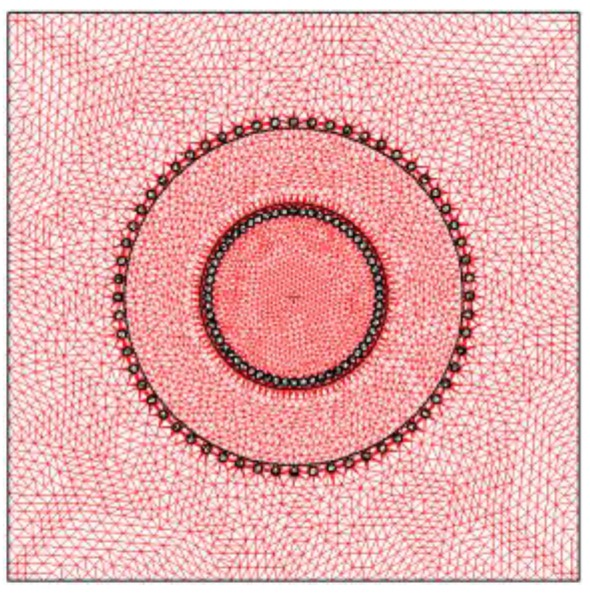
Mesh generated by the simulation program for the domain designed in 2D.

Next we choose the appropriate solver to perform the simulations. We carry out our 3D and 2D numerical simulations with the magnetostatic solver of the Ansoft Maxwell software as we have observed experimentally that the inductance varies little with frequency. Once this is done, we design the 3D and 2D computational domains (spatial regions that include the background of the domain, geometry of the core, winding, and coil former, if present). The real inductor consists of a toroidal core and one winding of copper wire. Figure 1a shows the built inductor. The 3D design is equivalent to the real geometry. For the 2D simulations we propose a cross-section of the real geometry, including the winding (Figure 1b and Figure 2), as an inductor model. Next, we assign the magnetic properties of the materials (relative permeability of the boundary, relative permeability and conductivity of the copper winding, relative permeability and conductivity of the coil former, if present, and the *B-H* curve of the ferrite). We also assign the boundary conditions, the ranges of the excitation current *I* (also called parametric intensity) and the necessary parameters to generate the meshing. To generate the mesh in 2D we have chosen an adaptive refinement of the mesh which consists of making a finer mesh at the spatial points that are more irregular, such as corners, regions with irregular borders, *etc*. This technique reduces the computing time and the convergence and tolerance. This algorithm is implemented into the Maxwell program. We then specify the parameters related to the adaptive analysis to generate the mesh: percent refinement per pass, the number of requested passes to stop the algorithm and the percent error (τ).

In the *simulation step,* we introduce the data of the premodeling into the Ansoft Maxwell program both in 2D and 3D and execute the program.

In the *postmodeling* step, for each value of *I* we obtain the values of the spatial distribution of the **B** and **H** fields that are an output of the simulation program.

We compute *B* by numerical integration of the module of **B** over the whole 3D volume of the core (or surface in the case of 2D) for each value of *I*,

(4) $$B=\frac{\underset{core}{∭}\left|B\right|\;dV}{V_{core}}$$

where

(5) $$V_{core}=\underset{core}{∭}dV$$

is the volume of the core.

From these values we compute the *B-I* curve and then we derive the *Φ-I* curve with the following equation,

(6) $$\Phi \approx N_{s}BA_{e}.$$

Finally the results in 2D are validated by comparing them with measurements and the simulation in 3D when there is convergence.

## 3. Experimental and Modeled Results

In this section we will first focus on describing how we carry out the simulating process and then go on to commenting how we carry out the study and validation.

**Figure 3:**
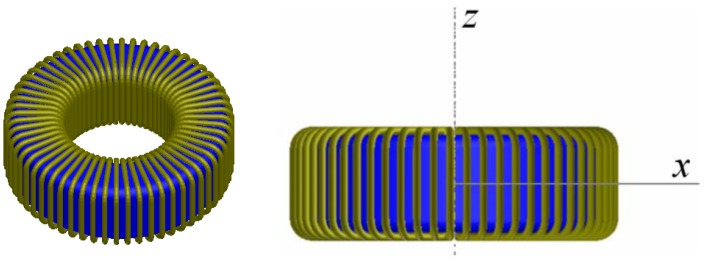
Geometry used for the simulations of the TN23/14/7 toroid with rounded corners and 60 turns.

We carried out 3D and 2D simulations by means of the Ansoft Maxwell magnetostatic solver on a personal computer. In order to perform the study we chose a toroidal inductor that consists of a TN23/14/7 ferrite core from the manufacturer Ferroxcube with a 60-turn winding of copper wire. We show the real inductor in Figure 1a. For the 3D simulations of this inductor we use two different geometries. The first geometry has rounded edges in order to represent the real inductor faithfully (see Figure 3) and the *x*, *y*, *z* coordinate system is centered in the geometric center of the core. We use a cylindrical domain with dimensions *D* (*r*,*z*) ∈ [0, 60] × [−40, 40] measured in millimeters and carry out the simulations with τ = 1% and a DC excitation current value *I* = 1 A (saturation region). After running the program for approximately one hour we notice that it is not able to reach a solution showing the message “error in building initial triangulation. Detected an error in the acis faceting”, indicating the non-convergence of the procedure. We repeat the numerical simulations with different bigger cylindrical domains and with different DC current values (*I* = 0.01, 0.02, 0.16, 3, 8 and 10 A). The first two values are in the linear region of the *Φ-I* curve, the third value is the intermediate region (knee of the curve) while the three last values are in the saturation region. Convergence was not reached with any of these values either. Once we finish the 3D simulations we perform a set of 2D numerical simulations under the same conditions for comparison purposes. To do so, we use a cross-section of the real inductor as a model included in a square computational domain achieving convergence in all cases. In Figure 1b we show the cross-section of the inductor and in Figure 2 we show the corresponding square computational domain including the triangular mesh generated by the program in the 2D simulations.

**Figure 4:**
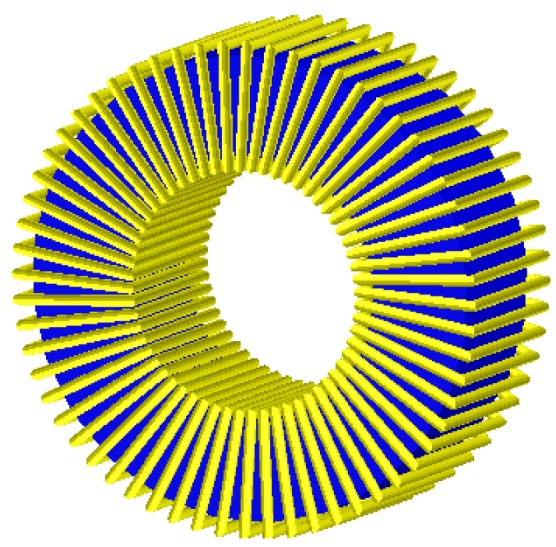
Geometry of the 60-turn inductor used for the 3D simulations with the TN23/14/7 ferrite core with right angled edges.

We carry out a second series of numerical experiments with the same values as before but with right-angled edges (see Figure 4), achieving convergence.

The validation of the design of the 2D domain and the analysis of the inductor behavior has been carried out on the basis of:

(a) Convergence and computational cost;
(b) The distribution of the |**B**| and |**H**| fields on the surface and along the cross-section of the ferrite;
(c) The *Φ-I*, *L-I*, *B-H* and *μ_r_-H* curves.

For (a) and (b) the 2D and 3D simulation data were compared to each other and in (c) the experimental results were included to validate our methodology.

### 3.1. Convergence and Computational Cost; Comparison of the 2D and 3D Simulations

Table 1 is a representative example of our results. We show the computational cost (computation time and number of finite elements) for different values of *I*, representative of the linear (*I* = 0.01 A), intermediate (*I* = 0.16 A) and saturation regions (*I* = 10 A).

**Table 1:** Computational cost for the 2D and 3D simulations with τ = 1%.

Current	CPU time		Elements	
I (A)	2D (s)	3D	2D	3D
0.01	8	24 min 16 s	6162	289,541
0.16	12	1 h 26 min	6162	289,541
10	8	46 min 7 s	6162	289,541

We can see that the computation time in 3D in the linear region is always less than the time required in the intermediate and saturation regions. The computation time in 2D is of the order of seconds while in 3D it is between 30 to 80 min. The number of needed elements for convergence is less than 7000 for 2D and over 289,000 for 3D. As another characteristic, for this specific case the number of elements is practically constant for all values of *I* both in 2D and in 3D.

### 3.2. The Distribution of the |***B***| and |***H***| Fields on the Surface and along the Cross-section of the Ferrite

**Figure 5:**
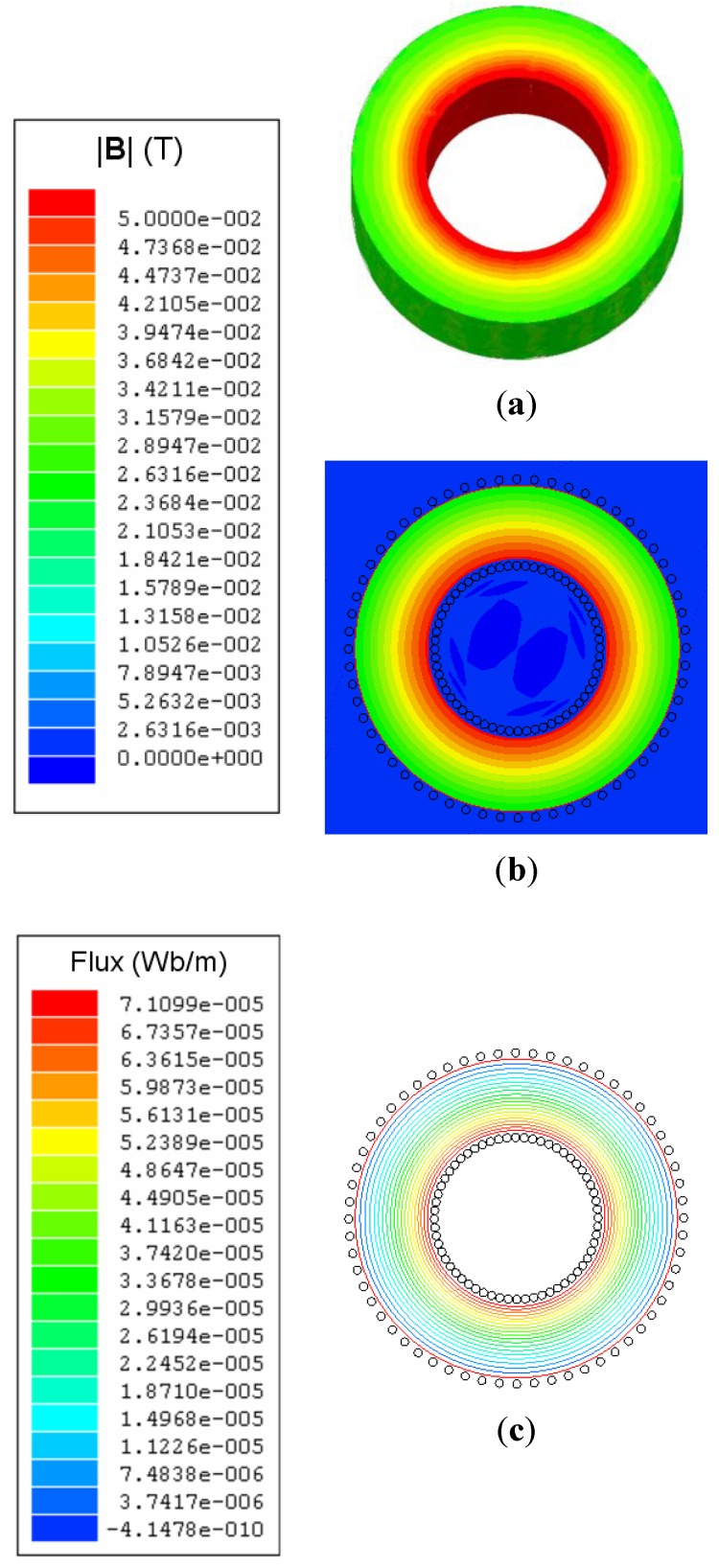
Geometry of the 60-turn inductor used for the 3D simulations with the TN23/14/7 ferrite core with right angled edges.

Figure 5a,b shows the distribution of the magnetic field |**B**| on the surface of the 2D and 3D cores for a current intensity *I* = 0.01 A (linear region). In Figure 5c we show the flux lines of the **B** field. We can see that both in 2D and 3D in the linear region the magnetic field is not distributed homogeneously in the core, varying from 0.05 T at the inner edge of the toroid up to 0.02 T at the outer edge, being approximately zero outside the ferrite core. As can be seen in Figure 5, the **B** lines are closed (∇**B** = 0) and in a circular shape centered on the axis of the core and contained inside of it because the magnetic permeability of the ferrite is very high compared to the one of vacuum (*μ_r_* >> 1).

**Figure 6:**
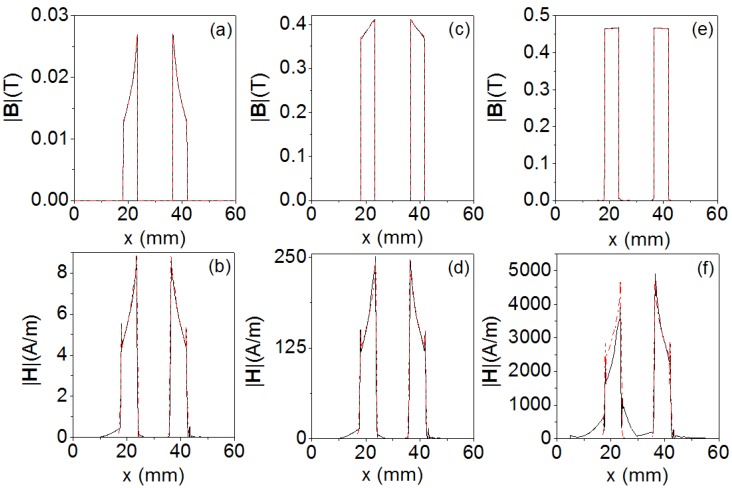
Moduli of the **B** and **H** magnetic fields as a function of the distance from the center of the inductor core (*x* = 30 mm) obtained by 2D (red dashed line) and 3D (black solid line) simulations, for (**a**,**b**) *I* = 0.0057 A (linear region); (**c**,**d**) *I* = 0.16 A (intermediate region); (**e**,**f**) *I* = 3 A (saturation region).

**Figure 7:**
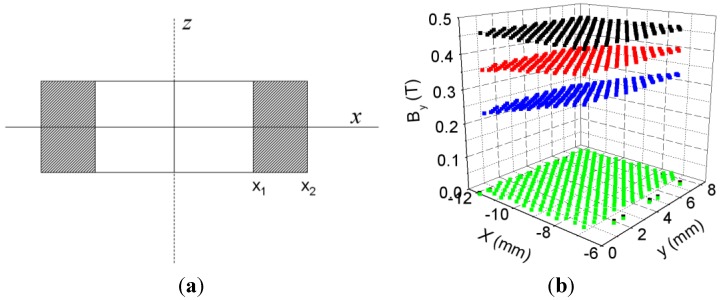

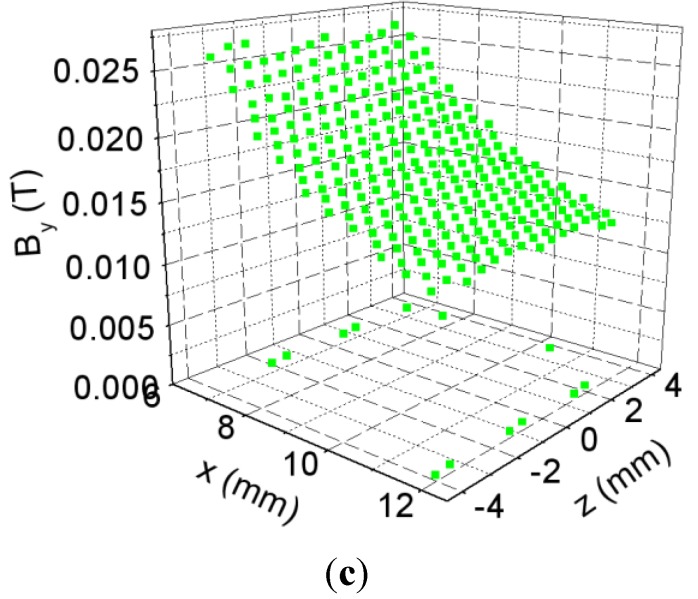
(**a**) Cross-section of the core in the *xz* plane; (**b**) Spatial distribution of *B_y_* in the *xz* section of the core for *I* = 0.0057 A (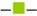), 0.16 A (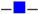) and 3 A (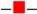) obtained by 3D simulations from *x*_1_ = −6 mm to *x*_2_ = −12 mm and (**c**) spatial distribution of *B_y_* in the *xz* section of the core for *I* = 0.0057 A obtained by 3D simulations from *x*_1_ = 6 mm to *x*_2_ *=* 12 mm.

In Figure 6 we plot the moduli of the **B** and **H** field as a function of the distance from the center of the core (*x* = 30 mm), obtained by 2D and 3D simulations for three values of the current intensity; *I* = 0.0057 A (linear region), *I* = 0.16 A (intermediate region) and *I* = 3 A (saturation region). In Figure 7 we show the spatial distribution of *B* ≈ *B_y_* in the *xz* plane of the core (see Figure 7a for dimensions) for different values of the current intensity (linear, intermediate and saturation).

In Figure 7 we show the spatial distribution of *B_y_* in the *xz* section of the core (see Figure 7a for dimensions) for *I* = 0.0057 A (linear region). Both in 2D and in 3D the values of |**B**| are similar, whereas there are discrepancies in the |**H**| field probably due to the edge effects in the case of saturation of the core. We can see clearly that the moduli of the **B** and **H** fields increase as the excitation current increases. The discontinuities observed in |**B**| in both 2D and 3D correspond to the interface between two different media (ferrite-air). Most of the **|B**| and |**H**| fields are confined inside the core; nevertheless we can observe some values of |**H**| outside the core. In Figure 7 we observe that in the spatial distributions in 3D there is scarcely any variation of the *B* field in the direction of the *z* axis, which justifies the 2D model validation in the *xy* plane. The distribution of the |**B**| field does not have the same shape for all values of the current intensity. In the case of an excitation value in the linear region, the |**B**| field decreases from the inside to the outside of the inductor from 0.028 T to 0.012 T. For a value in the intermediate region, the |**B**| field decreases from 0.4 T to 0.35 T.

Nevertheless, in the case of saturation the magnetic field is uniform, reaching a value of 0.46 T. In the case of |**H**| the shapes of the curves are very similar for all values of current intensity obtaining the highest values inside the core. These curves reach a maximum at the internal edge of the core and decreasing toward the outer edge as 1/r according to Ampère’s law.

The values change from 9 A/m to 5 A/m in the linear region, from 250 A/m to 125 A/m in the intermediate region, and from 4500 A/m to 2500 A/m in the saturation region. We noticed that the spatial shape of |**B**| in the linear case is practically the same as that of |**H**|. The maximum value (0.028 T) occurs at the inner edge and decreases toward the outer edge of the core where it reaches a lower value (0.012 T).

### 3.3. Experimental Validation of the 2D and 3D Simulations

First, we present and validate the flux curves and the corresponding inductances, and once this is done we show the *B-H* curves and their corresponding permeabilities.

**Figure 8:**
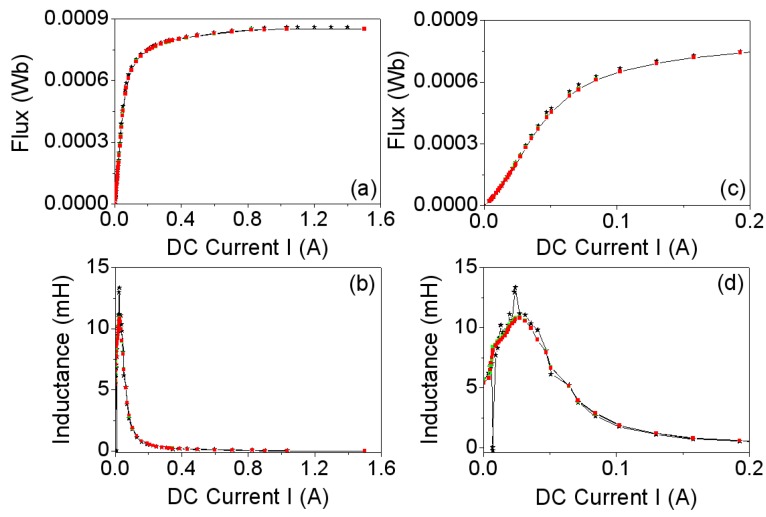
(**a**) *Φ*-*I* curves; and (**b**) *L*-*I* curves. Experiment (−*−). 2D (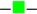) and 3D simulations (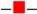); (**c**,**d**): Details of (a) and (b) in the range *I* ∈ [0, 0.2 A].

In Figure 8a we show the *Φ_exp_* curve (experimental magnetic flux) and the *Φ_mod_* curve (simulated magnetic flux) and in Figure 8b we plot their corresponding inductances (*i.e.*, derivatives $d\Phi_{exp}/dI$,$d\Phi_{mod}/dI$).

In Figure 8a we observe a good agreement between the *Φ-I* curves obtained by 2D and 3D simulations and by experiment. The same happens with the *L-I* curves. Both the magnetic flux and the inductance exhibit a nonlinear relation to the DC excitation current applied to the core. The saturation values tend to 0.0008 Wb, obtaining similar values in 2D, 3D and experiment. The inductance curves *L_exp_* and *L_mod_* reach a maximum of 11.8 mH at around 0.03 A which correspond to the inflexion points of the *Φ_exp_* and *Φ_mod_* curves. For values of *I* > 0.03 A the inductances decrease and tend to a very small final value with increasing *I*.

Regarding the validation of the *B-H* curves and their corresponding permeabilities, as commented on in Section 2, we obtain as the simulating step output the distribution of the **B** and **H** fields over the inductor for each value of current intensity *I*. Then, by numerical integration over the whole volume of the core, we obtain a pair of values (*B*, *H*) for each value of *I*, from which we obtain the *B-H* curve which agrees with the experimentally measured curve. Both in 2D and in 3D, we compute the (*B-H)_mod_* curves by numerical integration of *B* and *H*, that is to say, of the moduli of the **B** and **H** vectors in the whole volume or surface of the core using the Equations (4) and (5).

**Figure 9:**
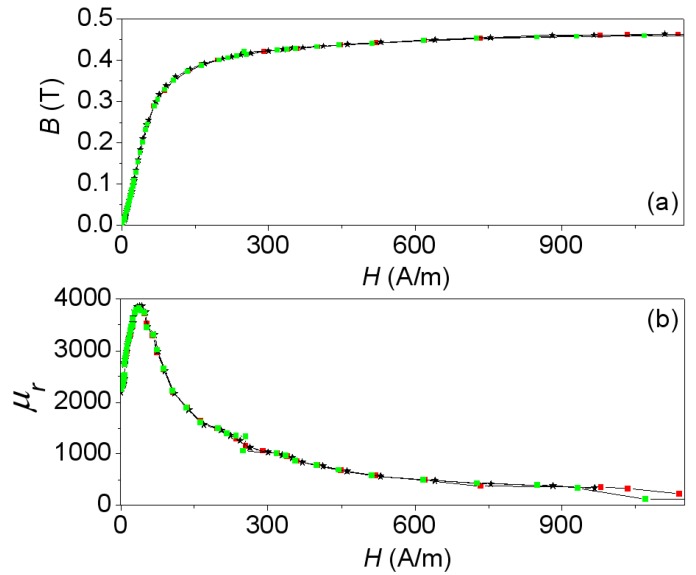
(**a**) *B-H* curves; and (**b**) *μ_r_*-*H* curves. Experiment (−*−) and 2D simulations (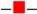) and 3D simulations (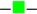).

In Figure 9a we show the experimental curve (*B-H)_exp_* together with the simulated one (*B-H)_mod,_* where it can be seen that there are three regions in the curve: the linear region at low current intensities (left side of the curve), the intermediate region (knee of the curve), and the saturation region at high current intensities (right horizontal region). Finally Figure 9b shows a plot of the relative permeabilities $\mu_{r\;exp}$ (experimental) and $\mu_{r\;mod}$ (modeled) computed from the above curves by the equation $\mu_{r}=B/\left(\mu_{0}H\right)$ and expressed as a funtion of the *H* field. The *B-H* curve of Figure 9 obtained experimentally is the one we have used at the premodeling step to obtain the results.

There is good agreement between the results obtained from measurements and from simulations. By using a cross-section of the real model of the inductor as a domain in the 2D simulation, we are able to reproduce the magnetic fields with the same precision as in 3D in the whole working regions of the ferrite (linear, intermediate and saturation). In the same way as between the magnetic flux and the inductance, the relation between *B* and *H* as well as the relative permeability and *H* is nonlinear. *B* tends to the saturation value *B_s_* ≈ 0.45 T as *H* increases. This saturation value is consistent with the value detailed by the Ferroxcube manufacturer in its catalog for this ferrite material (3F3). The permeability *μ_r_* reaches its maximum *μ_r_* ≈ 3900 at *H* ≈ 38 A/m and the permeability decreases until it reaches a saturation value of 200 with increasing *H*. These results allow us to affirm that practically all of the magnetic flux is confined inside the magnetic core.

## 4. Conclusions

In this paper we have proposed a procedure to compute the inductance of a ferrite inductor with a toroidal core in 2D, and have used the same procedure in 3D to compare the results. We have achieved convergence for all the excitation current values in both 2D and 3D. In order to achieve convergence, it was necessary to perform the 3D simulations with right-angled, instead of rounded, edges. There was good agreement between the results obtained by experiment and by simulations. By using a cross-section of the real inductor as a computational domain in the 2D simulations, the magnetic fields, flux and the inductance were reproduced with the same precision as in 3D in all working regions of the ferrite (linear, intermediate and saturation regions). We obtained the same spatial distributions of the *B* and *H* fields as the real inductors simulated in 3D except for slight deviations in the saturation region. The relation of the flux and inductance to the DC current applied to the inductor is nonlinear. The same relation appears between the *B* and *H* fields and between the relative permeability and *H*, both in the experiment and in the simulation.

As a future work we propose including the resulting inductance in a commercial circuit simulator to obtain the voltage and current waveforms of the inductor.
